# Infant Cereals: Current Status, Challenges, and Future Opportunities for Whole Grains

**DOI:** 10.3390/nu11020473

**Published:** 2019-02-23

**Authors:** Michelle Klerks, Maria Jose Bernal, Sergio Roman, Stefan Bodenstab, Angel Gil, Luis Manuel Sanchez-Siles

**Affiliations:** 1 Department of Research and Development, Hero Group; 30820 Alcantarilla, Murcia, Spain; michelle.klerks@hero.es (M.K.); mjose.bernal@hero.es (M.J.B.); 2 Marketing Department, Facultad de Economía y Empresa, University of Murcia, 30100 Murcia, Spain; sroman@um.es; 3 Department of Innovation, Hero Group, 5600 Lenzburg, Switzerland; stefan.bodenstab@hero.ch; 4 Department of Biochemistry and Molecular Biology II, School of Pharmacy, University of Granada, 18071 Granada, Spain; agil@ugr.es; 5 Institute of Nutrition and Food Technology “José Mataix”, Center of Biomedical Research, University of Granada, Avda. del Conocimiento s/n. 18016 Armilla, Granada, Spain; 6 Instituto de Investigación Biosanitaria IBS. GRANADA, Complejo Hospitalario Universitario de Granada, 18014 Granada, Spain; 7 CIBEROBN (CIBER Physiopathology of Obesity and Nutrition), Instituto de Salud Carlos III, 28029 Madrid, Spain

**Keywords:** infant cereals, infant food, whole grains, complementary feeding, health

## Abstract

Infant cereals play an important role in the complementary feeding period. The aim of this study was to review existing research about the quantity, type, and degree of infant cereal processing, with a special focus on whole grain infant cereals. Accumulating evidence shows many benefits of whole grain consumption for human health. Likewise, consumers are frequently linking the term whole grains to healthiness and naturality, and sustainable food production becomes a more important aspect when choosing an infant cereal brand. Whole grain cereals should be consumed as early as possible, i.e., during infancy. However, there are several challenges that food manufacturers are facing that need to be addressed. Recommendations are needed for the intake of whole grain cereals for infants and young children, including product-labeling guidelines for whole grain foods targeting these age stages. Another challenge is minimizing the higher contaminant content in whole grains, as well as those formed during processing. Yet, the greatest challenge may be to drive consumers’ acceptance, including taste. The complementary feeding period is absolutely key in shaping the infant’s food preferences and habits; therefore, it is the appropriate stage in life at which to introduce whole grain cereals for the acceptance of whole grains across the entire lifespan.

## 1. Introduction

Cereals, also called grains, refer to the crops that are harvested for dry grain only [1] and belong to the Gramineae family of grasses [2]. They include maize, rye, sorghum, millets, wheat, rice, barley, oats, and teff. Pseudocereals as amaranth, quinoa, and buckwheat are often included within the true cereals, because of their similar nutritional profiles and uses [3]. Cereal grains represent the most important source of the world’s total food [2,4]. Infant cereals are defined as “processed cereal-based foods” that are divided into “simple cereals which are or have to be reconstituted with milk or other appropriate nutritious liquids”; or “cereals with an added high protein food which are or have to be reconstituted with water or another protein-free liquid” [5,6].

**Figure 1:**
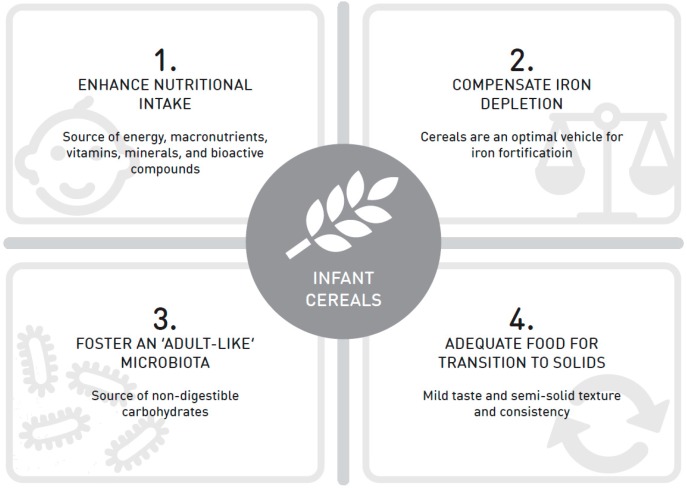
Four reasons to provide infant cereals as one of the first foods during the complementary feeding period.

In many countries, infant cereals are among the first foods that are introduced at the beginning of the complementary feeding period [7,8,9,10]. The choice to provide infant cereals as the first food during weaning can be explained for several reasons (see Figure 1). (1) Cereals are an excellent source of energy, which is very important at the age of six months when exclusively breastfeeding is no longer sufficient to cover the nutritional requirements of the infant [11]. Moreover, cereals provide a substantial amount of carbohydrates (starch and fiber) and proteins, but are also a source of vitamins, minerals, and bioactive compounds [12]. (2) Cereals are an optimal vehicle for iron fortification [13,14]. Therefore, the provision of infant cereals is effective at the beginning of complementary feeding, when the infant’s iron stores are depleting [15]. (3) Cereals provide non-digestible carbohydrates, which are mainly responsible for the development of an ‘adult-like’ microbiota by increasing the *Bacteroides* population [16]. During weaning, clear changes in the infant’s gut microbiota have been observed upon the addition of either wheat, sorghum, rice, or oats into a large intestine in vitro [17], and a higher proportion of complex carbohydrates in infant cereals has been shown to lead to a higher fermentative activity of the intestinal microbiota of infants aged six to 10 months in vivo [18]. (4) Cereals have a mild taste and a semi-solid texture and consistency, which is adequate for the transition from milk toward the acceptation of solid foods at the beginning of complementary feeding [19,20].

Despite the important role of cereals in infants and young children, still no consensus has been reached among pediatric organizations regarding adequate cereal intake, type of cereals, and degree of cereal processing (whole grain versus refined cereals). Our objective is to shed more light on these issues by reviewing existing research regarding the quantity, type, and degree of cereal processing. Even though we acknowledge the literature on cereals for adults, the focus of this article is on infant cereals, with special emphasis on whole grain infant cereals. Indeed, this paper is organized as follows. First, we review existing recommendations for infant cereal intake. Then, we focus on whole grain cereals and compare them to refined cereals in terms of their impact on infant nutrition and health. Finally, challenges for the food retailers, manufacturers, and policy makers are discussed, and future opportunities that can be derived from these challenges are proposed for infant cereals.

## 2. Recommendations for Infant Cereal Intake

Nutritionists’ advice for complementary feeding has changed dramatically over the last decade, as nutrition science has progressed and demonstrated the importance of good nutrition during the months following the period of exclusive breastfeeding. For example, while in the past the avoidance of allergens was the standard recommendation, today, one would rather recommend early introduction in small steps, while still breastfeeding. In many areas, no consensus has been reached yet. Likewise, recommendations for cereal intake in infant and young children vary across countries, and they often remain vague. There seems to be only one consensus: the intake of cereals is not recommended before the age of four months. Examples of existing recommendations for cereal intake in infants and young children are provided in Table 1. In Europe, the European Society for Paediatric Gastroenterology, Hepatology and Nutrition (ESPGHAN) recommends introducing iron-rich complementary foods such as cereals from four months alongside breastfeeding [15,21]. Similarly, The European Food Safety Authority (EFSA) recommends introducing iron-rich food after four to six months of age [22]. However, in the United States (US) and New Zealand, cereal intake before the age of six months is discouraged [23,24,25,26]. Furthermore, countries communicate their cereal intake advice via the number of servings [25,26,27], tablespoons [28], or exact grams [29,30], while in Europe and the US, there are also organizations that do not define specific amounts [15,21,22,23,24,31].

Some countries only provide recommendations about whether to introduce cereals with or without gluten, yet no general agreement has been reached about this issue. For example, France and Spain advise providing gluten-free cereals before five to six months of age [31,32], whereas the ESPGHAN indicate that gluten may be introduced between four and 12 months of age [21] (Table 1). Generally, information about the type of cereals is not incorporated within a country’s recommendations. It seems that the type of cereals given to infants might be influenced by cultural beliefs. In the United Kingdom (UK) and Ireland, infant rice cereals are the most popular first complementary food [33,34,35]. In the Nordics and Baltics, in many instances, oats are the infant’s first solid introduction [36,37], except for Norway, where maize/rice infant cereals are the most common weaning foods [38]. In Spain, wheat or rice are the most consumed infant cereals [39]. According to the Feeding Infants and Toddlers Study (FITS) in the US, the most common food sources of starch in infants and young children are iron-fortified infant cereals, which are primarily made of rice or oats [13,40]. Likewise, infant cereal intake in Canada is mainly rice-based [41], but mothers seem to start with maize porridges in African countries [42,43,44]. On a final note, it is often unclear to which extent the cereals used for the formulation of infant cereals are recommended to be processed (refined or whole grain). The 2015–2020 Dietary Guidelines for Americans encourages the consumption of nutrient-dense foods such as whole grains in children aged between one and 18 years (1.5 to four ounce-equivalents) to increase dietary fiber, ensure normal gastrointestinal function, and prevent chronic diseases [45]. In the next section, we will describe the reasons in favor of the use of whole grains in infant cereals. 

## 3. Reasons to Believe in the Use of Whole Grains in Infant Cereals

According to the American Association of Cereal Chemistry International (AACCI) [46]: “Whole grains shall consist of the intact, ground, cracked, or flaked caryopsis, whose principal anatomical components—the starchy endosperm, germ, and bran—are present in the same relative proportions as they exist in the intact caryopsis”. The HEALTHGRAIN Consortium of the European Union added in 2010 to this definition that cover removal and small grain component losses are allowed, but they should be less than 2% of the grain or less than 10% of the bran [3].

This chapter discusses why the use of whole grains for infant cereals would be better from a nutritional, health, and consumer point of view, compared to the use of refined cereals.

### 3.1. Nutritional Differences between Whole Grains and Refined Cereals

All grains are made up of three parts: the multi-layered outer fiber-rich bran, the micronutrient-rich and lipid-rich germ, and the starchy endosperm. While in whole grains, all components of the grain are still present (80–85% endosperm, 10–14% bran, and 2.5–3% germ), refined cereals consist only of the endosperm [12].

The highest proportions of compounds such as fibers, vitamins, minerals, and other phytochemicals are found in the bran and germ of the grain. Several biological compounds have been described in whole grain cereals with interesting physiological functions (e.g., immune system stimulation, cell signaling and/or gene regulation, antioxidant, anti-inflammatory) and potential protective mechanisms (e.g., diabetes, cancers, cardiovascular diseases) [12]. The major bioactive compounds in whole grain are vitamins, minerals, and phytochemicals including phenolics, carotenoids, vitamin E, lignans, β-glucans, inulin, resistant starch, sterols, and phytates [47]. Although these bioactive compounds are present in whole grains in general, some bioactive compounds are specific to certain cereals such as γ-oryzanol in rice, avenanthramide, avenacosides, and saponins in oats, β-glucans in oats and barley, and alkylresorcinol in rye [12]. Therefore, processing whole grain cereals to refined cereal products leads to major losses of these protective compounds, as they lack the bran and germ fractions [12]. For example, it has been shown that after refining wholemeal flour into white flour, only 42% of fiber, 17% of magnesium, 21% of zinc, 8% of selenium, and 21% of vitamin E were retained [48]. Also, a large drop in phenolic compounds (an important group of the phytochemicals) has been observed after milling the whole kernel of maize [49], and a review by Ktenioudaki et al. (2015) indicated that milling caused a decrease of phenolic compounds, flavonoids, tocols, carotenoids, and sterols in several cereals [50]. Results reported by Adom et al. (2005) revealed that the majority of phytochemicals in whole wheat grain are present in the bran and germ. The content was found to be 15 to 18-fold higher in the bran and the germ compared to the content in the endosperm [51]. Accordingly, it has been shown that analyzed phenolic compounds were enriched in the bran of the rye kernel [52], relatively higher in the bran of wheat [53], and more abundant in whole wheat compared to refined samples of wheat [54]. It seems that especially the aleurone layer of the bran contains a high level of bioactive compounds [55] that have a high antioxidant and anti-inflammatory capacity [56]. It can be concluded that whole grains are more nutrient-dense compared to refined cereals [57]. To illustrate, the nutritional differences between whole wheat flour and refined wheat flour are shown in Table 2.

One main compound that is removed during the refining of cereals is dietary fiber. Dietary fibers (e.g., β-glucans, arabinoxylans, resistant starch, and inulin) are a major contributor to several health benefits [58,59]. Fiber, defined by the EFSA as “non-digestible carbohydrates plus lignin” [60], has according to Directive 2008/100/EC “beneficial physiological effects such as: decreasing intestinal transit time, increasing stool bulk, fermentable by colonic microflora, reducing blood total cholesterol levels, reducing post-prandial blood glucose, or reducing blood insulin levels” [61]. The health effects of fibers depend on the degree of fermentation. Traditionally, fibers can be classified into insoluble and soluble fibers. Soluble fibers are highly and rapidly fermented by the microbiota, while insoluble fibers are poorly and slowly fermented. Soluble fibers increase viscosity, bile acid excretion, serum lipids, and short-chain fatty acids, which benefit post-prandial glucose response, among others. Insoluble fibers are capable of absorbing water, and favor laxation and intestinal regulation [12,62]. The ratio of soluble to insoluble fiber is different depending on the type of cereals (e.g., wheat 1:5, oats 1:1.5) [12,63].

Whole grains, as a rich source of dietary fibers and other bioactive compounds, may modulate the gut microbiota, and therefore impact consumers’ health. While extant research has focused on the contribution of whole grains to health, little research has been conducted on how their dietary fibers and other constituents from whole grain matrices affect the gut microbiota. Oats may have particular effects on the gut microbiota in comparison with other grains, due to their high levels of soluble fiber (mainly of β-glucan) [64]. In fact, there is controversy regarding how different types of whole grains can affect gut microbiota. On one hand, it has been shown that whole grain wheat breakfast cereal has a prebiotic effect on the human gut microbiota compared with wheat bran [65]. However, the intake of whole grain and fiber-rich rye bread versus refined wheat bread did not differentiate intestinal microbiota composition in adults with metabolic syndrome [66]. Moreover, short-term consumption of whole grains (six weeks) increased stool weight and the frequency of bowel movements, but had modest positive effects on gut microbiota compared with refined grains [67]. Similarly, a six-week intervention with whole-grain rye and wheat in healthy overweight adults affected some markers of gut health without altering the fecal microbiota [68]. Nevertheless, soluble feruloylated arabinoxylan oligosaccharides and polyphenols isolated from rice bran have been shown to have positive impacts on human gut microbiota through a prebiotic function [69].

However, the proposed health benefits of whole grains go beyond the effects from dietary fiber only. Considering the huge number of components that are involved in whole grains, it is likely that they have synergistic effects in contributing to the potential health benefits according to the holistic approach described by Fardet (2014). The author reported that the effects of nutrients depend on a whole food or matrix, meaning that the effects of food as a whole are different than the sum of its individual compounds [70].

### 3.2. Benefits of Whole Grain Consumption

Accumulating evidence demonstrates that the consumption of whole grain foods has several benefits for human health [12]. More specifically, whole grain consumption has extensively been shown to reduce weight gain and the risk of obesity [72,73,74], type 2 diabetes [75,76,77,78,79], (colon/colorectal) cancer [80,81,82,83], and cardiovascular diseases [82,84]. Furthermore, whole grain consumption reduces the risk of respiratory diseases, infectious diseases, and all-cause and cause-specific mortality [82,85,86,87,88]. Recently, whole grain intake has also been linked to an improved cognitive function in adults [89]. Several mechanisms that are induced by the intake of whole grains could explain these protective effects. These mechanisms comprise the reduction of inflammatory processes [74,90,91], the enhancement of insulin response [79,92,93], the modification of blood lipid profiles [92,94,95], and improvement and maintenance of the gut health [67,68,96,97,98]. Observational evidence showing the health benefits of whole grain consumption is consistent; however, results from randomized controlled trials have not been as convincing as those from observational ones yet [77,99].

Although the health benefits of whole grains in adults are broadly acknowledged, the question arises of whether whole grain consumption could have the same or even larger benefits in infancy and early childhood. Unfortunately, only a limited number of empirical studies have been conducted in infants and children.

In a crossover clinical trial, systemic inflammatory biomarkers were evaluated of 44 overweight or obese Iranian girls aged between eight and 15 years old when half of their cereal intake consisted of whole grains. Changes in biomarkers such as C-reactive protein, soluble intercellular adhesion molecule-1, serum amyloid A, and leptin were found after six weeks of whole grain consumption. However, no significant effects of whole grain intake on the subjects’ weight and body mass index (BMI) were found [100]. A recent Danish cross-sectional study conducted by Damsgaard et al. (2017) on 713 children aged between eight and 11 years investigated the association between the amount of ingested whole grain and the type of whole grain with fat mass and biomarkers related with cardiometabolic risk profile [101]. The total intake of whole grains (median intake 52 g/day) was not correlated with fat mass index, but it was associated with serum insulin. The intake of whole grain oats specifically was inversely associated with fat mass index, systolic blood pressure, low-density lipoprotein (LDL) cholesterol, and insulin [101]. Another study by Koo et al. (2018) with 63 Malaysian children aged between nine and 11 years showed that an intervention of six 30-minute nutrition education classes emphasizing the consumption of whole grains and the substitution of whole grain foods on a daily basis during school break time over a 12-week period significantly resulted in lower BMI z-scores, lower body fat percentage, and a lower waist circumference compared to the children that did not received the intervention (control group). However, the exact amount of whole grains consumed per day that caused the achieved effects in this study was not reported [102]. Furthermore, in the prospective, randomized Special Turku Coronary Risk Factor Intervention Project (STRIP) with a sample of 941 children, dietary counseling was given biannually based on the Nordic Nutrition Recommendations, and one of the major aims was to promote the intake of vegetables, fruits, and whole-grain products, among other measures. Achieving the dietary targets during the 20-year dietary intervention was associated with better insulin sensitivity and serum lipid profile throughout the early life course [103]. Lastly, a clinical trial of 28 infants and children (median age 7.2 years) with chronic functional constipation showed that an intervention of dietician’s advice to follow a diet oriented according to the Food Guide Pyramid with an emphasis on fruits with peel, pulses, vegetables, seeds, nuts, and whole grain cereals resulted in improved bowel habits for 75% of the subjects. The authors concluded that a diet high in dietary fiber and bran is feasible in constipated children, and that it will ameliorate constipation [104], but more controlled clinical trials are needed in order to demonstrate the benefits of whole grain consumption for childhood constipation [105]. In summary, results from these studies conducted on children indicate that whole grain consumption might be beneficial for health at this age stage, as shown by e.g., the biomarkers related to obesity and cardiovascular diseases. This evidence is promising, but still, more research is needed to analyze the effects of whole grain intake in infants and young children.

Although the few randomized controlled trials have shown that whole grains might be beneficial in childhood, it seems that there is an urgent need to improve whole grain intake. Findings from several studies investigating the dietary patterns of children demonstrate that the consumption of whole grains in children across the globe (young children of 18 months up to adolescents of 18 years) is infrequent and poor [106,107,108,109,110]. However, this pattern seems to be stronger in Europe and Asia as compared to the US [110]. For example, three-day dietary records from 821 German children aged between two and 18 years showed that only about 4% of their total grain consumption consisted of whole grains (mean intake ranged from 20–33 g/day). No whole grain intake was observed in nearly 20% of all dietary records. This means that the German Food Guide Pyramid’s aim of 50% of total grain intake should consist of whole grains was by far not reached [106]. Another seven-day dietary survey was held among 1171 French children aged between three and 17 years old. More than half of these children reported to never consume any whole grains. Of those children who did, mean whole grain intake was between six and eight g/day and 13 g/day in children aged between three and 12 years and 13–17 years, respectively [107]. In the UK, based on four-day diet diaries of 1502 children between 1.5–17 years, it was found that 15% did not consume any whole grains. In this age range, a mean of roughly 18 g/day of whole grains was consumed, which is below the country’s recommendations of 32 g/day [108]. Lastly, whole grain intake of 561 Singaporean children aged between six and 12 years was assessed by 24-h recalls. Results showed that only 38% of the respondents indicated consuming whole grains during the data collection days, meaning that 62% did not. Median intake of whole grains was approximately 15 g/day. Six percent of all the children reached the set 48 g/day of whole grains, which is an amount that is most commonly associated with improved health outcomes [109].

### 3.3. Consumer Perspectives on Whole Grains

From a consumer perspective, the term “whole grains” is frequently linked to words such as “wholesome” [111], “healthy” [112,113], “with minimal processing” [112] and “natural” [111,112,113]. This is particularly relevant as a recent systematic review concluded that: “Food products that are not perceived as natural may not be accepted by the majority of consumers in most countries” [114] (p. 50). Also, young people perceive whole grain foods as healthy and somehow related to healthiness [115,116].

Jones and Sheats (2016) indicated that many new trend drivers are exerting more influence on consumer behavior and the consumption of food and grains than before. These trend drivers involve issues such as concerns about the environment, misconceptions of technologies and practices used in food manufacturing, and worries about the rising obesity rates [117] (p. 29). Sustainability issues are particularly relevant. For example, evidence from a systematic review by Nelson et al. (2016) showed that a dietary pattern higher in plant-based foods (e.g., vegetables, seeds, whole grains) and lower in animal-based foods is significantly associated with a minor impact on the environment [118]. Furthermore, recent findings from Román and Sánchez-Siles (2018) showed that environmental protection, and organic and sustainable food production play a major role in explaining infant cereal brand choice [119]. In this vein, it is important to consider that whole grain food products are less processed than refined grains, and more sustainable from an environmental point of view [70]. Next, we will focus on the challenges and future opportunities derived from the research reviewed earlier in this manuscript.

## 4. Challenges and Future Opportunities

A low intake of whole grains in adult populations might be explained by a number of reasons such as the difficulty of identifying whole grain foods [113], a lack of knowledge of how to prepare them [112,116], higher prices [112,113,116], unfamiliarity with whole grain products [113], limited availability, and poorer perceived taste and texture [112,113]. Similar evidence was found with younger consumers (11–16 years) [115]. Accordingly, food manufacturers face many challenges that need to be addressed, including a lack of unified recommendations for whole grain intake, consumer confusion when identifying whole grain food products, and the need to ensure the highest health and safety standards for whole grain infant cereals while retaining sensory appeal. In what follows, we elaborate on these challenges and discuss future opportunities derived from them, both for the food industry and policy makers.

### 4.1. Lack of Unified Recommendations for Whole Grain Intake

Emerging evidence of the health benefits of whole grain consumption emphasizes the need for recommendations to incorporate whole grain foods into the diet. Such recommendations have been increasingly added to the already existing dietary guidelines in several countries lately, and are strongly focused on the general population [62]. The recommended whole grain consumption for the general population is inconsistent, and varies from country to country. Where some organizations provide specific daily doses targets, others advise increasing whole grain consumption in general [120,121,122]. For example, the World Health Organization (WHO) recommends an “increase [in] consumption of whole grains”, but also states that “appropriate intake levels shall be determined in accordance with national dietary guidelines and considering cultural traditions and national dietary habits and practices” [120]. Another non-specific recommendation is given in the UK, where the National Health Service (NHS) states: “Where you can, choose whole grain varieties.” [123]. In Spain, it is generally recommended to choose whole grain bread, pasta, rice, and flour, as shown in their food pyramid [124]. Interestingly, in the US, it is recommended to increase whole grain intake by replacing refined grains with whole grains, but also “to consume at least half of all grains as whole grains” [45]. In Denmark, Sweden, and Norway, it is more specifically recommended to consume at least four portions/day, which is equal to 75 g of whole grains/day for a 2400-kcal diet, or 90 g/day for men and 70 g/day for women [125,126,127]. However, many countries across the globe, such as those located in Southeast Asia, do not have any whole grain recommendations in their dietary guidelines [128].

Detailed whole grain recommendations in terms of quantity for infants and young children were also absent until recently. Yet, the current ongoing debate has led to the intention of some organizations to carefully integrate whole grain recommendations for children under two years of age (Table 3). Currently, Spain recommends that half of the cereal intake should be whole grains in infants and young children younger than 24 months [129]. In Australia, recommendations are more amount-specific, and include recommending 16 servings/week and 19 servings/week of whole grains for young children aged between 13–23 months and 24–36 months, respectively. The National Health and Medical Research Council (NHMRC) defines one serving as 40 g of bread, which is equal to one slice, so the daily Australian recommendation for whole grain consumption can also be calculated as 90 g (two slices) and 110 g (three slices) of whole grain bread/day [27]. Multiple organizations in the US are recommending whole grain intake. From the ages of six to 12 months, it is recommended by Healthy Eating Research to offer the infant a variety of whole grain products, such as brown rice or whole grain cereals [130]. For infants and young children between 12–36 months old, it is recommended to start with two ounces cereals/day, starting with one ounce of whole grains at 12 months [131], and increasing intake to at least 1.5–2.5 ounces of whole grains/day at 36 months of age [45]. It is specified that in general, one ounce is equal to one slice of bread, one cup of ready-to-eat cereals, or half a cup of cooked rice, pasta, or cereals [45] (Table 3).

To increase whole grain intake, future strategies need to be explored and incorporated. In general, it seems that individuals around the world are confused about the amount of whole grains that should be consumed daily [132]. Also, the development of whole grain infant cereals within the food industry is hindered by a lack of clear regulations on whole grains. Clear global recommendations regarding daily whole grain intake, especially for infants and young children below two years of age, would provide a solid basis for the industry, healthcare professionals, and consumers to make accurate choices in processing, advising, and purchasing whole grain foods. 

### 4.2. Consumer Difficulty Identifying Whole Grains

Although whole grains have been defined by several organizations as discussed earlier and the whole grain stamp has been used on more than 9000 products in 41 countries [134], until now, a consistent global definition for whole grain foods has not yet been developed. Without a standard definition for whole grain foods, packaged whole grain food products might provide different amounts of whole grains per serving. To ensure that consumers can easily identify foods with high whole grain content, accurate and credible labeling is needed. In fact, a roundtable’s expert panel represented by individuals from Europe and the US proposed, aligning with both the Dietary Guidelines for Americans 2010 (DGA) and the approved AACCI whole grain characterization [135], that a food providing at least eight grams of whole grains per 30 g (27 g/100 g) is nutritionally meaningful, and can be considered a whole grain food [136].

However, the US Whole Grains Council (2014) was not convinced about this definition, because it does not refer to wet or dry weight, and thus it can be misleading to label a food as whole grain when the product might contain more refined grain ingredients than whole grain ingredients [134]. Therefore, Ross et al. (2017) offered, on behalf of the HEALTHGRAIN Forum, a new definition, namely: “A whole grain food is one for which the product is made with ≥30% whole grain ingredients on a dry-weight basis and contains more whole grain ingredients than refined grain ingredients”. When a product meets these requirements, it can be labeled as a whole grain food, and display the whole grain stamp with the accurate proportion of whole grain content. The authors acknowledged that, “This 30% is selected as a starting point for whole grain food labeling. There is insufficient evidence to state that 30% of a product is the significant amount for health and this definition does not aim to set thresholds for health claims” [137] (pp. 528–529).

Consumer knowledge about nutrition and the ability to understand product labels are important factors to consider. For example, findings from Violette et al. (2016) showed that 63% and 66% of older adults (aged > 65 years) correctly identified whole grain cereals and crackers, respectively; however, refined bread was incorrectly identified by 46% of the respondents as being whole grain [138]. Accordingly, more transparent and clear labels would make it easier for the consumer to identify and understand whole grain foods [139]. Furthermore, knowledge education regarding the health benefits in later life and the use of whole grain infant cereals in early life may cause consciousness and a change in consumer purchase behavior [136].

### 4.3. Safety and Health Concerns of Whole Grains and Infant Cereal Processing

Historically, mothers prepared cereals for infants themselves at home. These cereals were finely ground, toasted, boiled, and mixed with water or milk. This mode of preparation is still possible, but nowadays, it is easier if a blender or a food processor is used to improve the texture. Today, commercial infant cereals are commonly used. These cereals do not need to be ground nor cooked before consumption, and are, in most countries, produced according to strict safety regulations.

Since infants are a vulnerable group, safety and health challenges are of particular concern in the manufacturing of infant cereals. In this subsection, we will describe some examples of safety and health concerns regarding the content of arsenic and mycotoxins in whole grain raw materials, the production of contaminants such as acrylamide during processing, and how some processes applied in infant cereals will increase the levels of free sugars.

#### 4.3.1. Safety Concerns of Whole Grain Infant Cereals

The use of whole grains in infant cereals may come with some safety issues, because whole grains usually contain more contaminants than refined cereals. Two main reasons are described in a recent review published in this journal by Thielecke and Nugent (2018) [111]. Firstly, the outer layers of the cereal grain are more likely to be exposed to contaminants such as heavy metals and mycotoxins from the soil, such as arsenic or pesticides. Secondly, the germ and the bran contain higher concentrations of asparagine, which is an amino acid that leads to the formation of acrylamide during the processing of cereals [111].

Arsenic is a heavy metal with neurotoxic and carcinogenic effects. It can be present in high concentrations in rice, which is a common cereal used in the manufacturing of infant cereals. Since arsenic is accumulated in the outer layer of rice, the concentrations in whole grain rice cereals are higher compared to their refined counterparts [111,140].

The US Food and Drug Administration (FDA) and the European Commission Regulation established a maximum level of 100 µg/kg or 100 ppb of inorganic arsenic in rice used for foods targeted at infants and young children [141,142], and companies are striving to achieve lower values in infant cereals. Manufacturers select sources of rice and rice-derived ingredients with lower inorganic arsenic levels [141]. However, values of arsenic in infant cereals are sometimes still too high. This finding follows from a survey conducted in the US by Healthy Babies Bright Futures (HBBF) that analyzed the inorganic arsenic content in 105 infant cereals of leading brands. The results indicated an average arsenic content of 73 ppb in rice infant cereals and 96 ppb in whole grain rice (brown rice) infant cereals. The authors concluded that arsenic content in rice infant cereals is still excessive, and therefore infant cereals manufacturers need clearer strategies to reduce the arsenic in their products. The authors requested that the FDA recommendations limit the intake of rice infant cereals until mitigation of arsenic level has been reached. They proposed increasing the intake of multigrain or other cereals instead of decreasing the consumption of rice infant cereals [143].

Mycotoxins are toxic secondary metabolites produced by certain filamentous fungi (molds). Mycotoxins can accumulate in maturing cereals and other food and feed crops in the field and in grain during transportation [144]. In a recent study conducted in the US retail market, one or more mycotoxins were found in 69% (101/147) of the infant and toddler foods, and 50% (34/68) of the breakfast cereals. However, the concentrations of detected mycotoxins were lower than the current FDA action, and guidance levels and rice-based cereals appeared to be less susceptible to mycotoxin contamination than other cereal types [145]. Mycotoxins can be present in the outer layers of grains, but cleaning of the grain ensures a decrease of mycotoxins [111]. Furthermore, the bioactive compounds found in whole grains can protect against the negative impact that mycotoxins might have [111]. Manufacturers must carry out controls to limit the levels of mycotoxins according to the maximum levels for commercial infant cereals established by the European Commission 1881/2006 (aflatoxin B1: 0.10 µg/kg, ochratoxin A: 0.50 µg/kg, patulin: 10 µg/kg, deoxynivalenol: 200 µg/kg, zearalenone: 20 µg/kg and fumonisins: 200 µg/kg) [146].

Acrylamide, which is formed when toasting raw material, is another contaminant to consider in infant cereals due to its carcinogenic effects. The toasting of raw material is carried out to remove possible contaminants such as fungi, yeasts, bacteria, insect eggs, and the inactivation of the lipoxidases that are responsible for fat rancidity and improve the sensory characteristics of the final product. When the temperature and exposure time of toasting are high (usually above 120 °C) and the moisture content is low, toasting can stimulate the formation of acrylamide [147]. Although the asparagine (and thus acrylamide) content is higher in whole grains than in refined products, the overall higher health benefits of whole grains may outweigh this disadvantage [148,149,150]. In this sense, companies decrease the temperature and time of toasting, or use an enzymatic treatment with asparaginase for the mitigation of acrylamide formation during infant cereal processing to maintain levels below the established thresholds by the European Commission (2017) [151].

#### 4.3.2. Sugar-Related Concern in Infant Cereals

During the processing of infant cereals, more specifically when the infant cereals are enzymatically hydrolyzed, free sugars are produced. This should be considered, because the recommendations for infants and young children under the age of two are strict with regards to the intake of free sugars. The ESPGHAN states that no sugar should be added to complementary foods, and free sugars should be minimized or avoided [21].

However, many manufacturers, depending on the country, choose to enzymatically hydrolyze the cereals after toasting. The enzymatic hydrolysis of infant cereals is carried out for some reasons, including the technological aim to stabilize the viscosity of the infant cereals after preparation and the physiological aim to increase the starch digestibility. For a long time, there has been an incorrect belief that infants and young children are not able to digest or hydrolyze starch due to a low presence and activity of pancreatic α-amylase, which is the main enzyme that is responsible for starch digestion [152,153,154]. On the contrary, infants are able to digest starch even more efficiently than adults. There are two facts that contradict the old belief that infants are not able to digest starch. Firstly, other enzymes such as glucoamylase–maltase and salivary α-amylase make up for the physiological lower activity of pancreatic α-amylase [155,156,157,158]. Secondly, infants have a higher capacity to ferment the non-digested starch (resistant starch) that reaches the colon, which is also called energy salvage, compared to adults [159,160]. Therefore, it could be assumed that hydrolysis is an unnecessary step in the manufacturing of infant cereals.

### 4.4. Sensory Acceptability of Whole Grain Cereals

One of the potential advantages of refined cereals, compared to whole grains, is the improved sensory characteristics that are highly accepted by consumers [161,162]. Findings from a study conducted in Singapore among 21 to 26-year-olds largely confirmed the characterized barriers from studies carried out in Europe and North America that were discussed earlier in this article, indicating categories such as “sensory” and “habitual” as the most common barriers for whole grain consumption [163].

Interestingly, research has shown that acceptability increases with repeated consumption. For example, a noteworthy outcome of an intervention by Kuznesof et al. (2012) revealed that many participants “surprisingly” liked the taste of the whole grain foods when they were introduced to it, as they prejudged disliking the taste due to negative experiences from years ago. Frequent consumption resulted in participants that learned to like the whole grain foods [112]. This result was also found in two other studies, which showed that direct exposure to whole grains by including them in the diet tended to increase acceptability and consumption [163,164]. In other words, familiarization with whole grain foods is likely to reduce negative thoughts about the expected taste.

In either way, sensory appeal remains a key factor in food choice and (whole grain) intake [113,115]. In this light, the sensory liking of grain products seems to depend on the type of food product. A preference for refined pasta and rice was found over their whole grain counterparts, but both types of tortillas and bread were equally liked among college students [116]. Other studies indicated that the gradual and/or unknown replacement of refined grain ingredients with whole grain ingredients does not affect acceptability or consumption in young adults [165] and schoolchildren [166,167,168]. Recent findings from the qualitative and quantitative study by Román and Sánchez-Siles (2018), on a sample of parents of infants and young children under 18 months, highlight the importance placed on the sensory properties (with special emphasis on taste and texture) when buying infant cereals [119]. In this regard, a study by Haro-Vicente et al. (2017) tested whether infant cereals containing 30% whole grains would be similarly accepted by both parents and infants/young children aged between four and 24 months compared to the same cereal with only refined grains. Other attributes such as color, smell, and taste were also evaluated by the parents. Importantly, results of the eight-day experimental trial showed that among infants, young children, and their parents, the sensory acceptability of infant cereals with added and without added whole grains was found to be the same. Furthermore, there were no significant differences experienced between the two infant cereals for any of the other attributes [169]. All of the main findings of studies evaluating the sensory acceptability of whole grains are summarized in Table 4.

The eating behaviors of infants and young children eating behavior is influenced by both intrinsic (e.g., genetics/predisposed biological tendencies, age) and environmental (e.g., parents, demographics) factors [170]. Reflecting their basic biology, infants have an innate preference for sweet taste and a dislike for bitter taste [171]. The substitution or addition of whole grains might cause an adverse taste [162] due to for example phenolic compounds [172] and oxidation changes of linoleic acid [173], which will possibly be rejected by infants. Therefore, parents and caregivers play a critical role in shaping infants’ and young children’s dietary patterns during complementary feeding, which is the time when food preferences, eating skills, and habits are grounded [174,175,176,177]. Importantly, the eating habits that are established during this stage might become a potential barrier for future consumption [163]. Even the foods that might be rejected primarily may be learned to be liked and consumed by children through repeated exposure, associative conditioning, or in interaction with contextual signals from the eating environment. Infants and young children have the ability to learn to like foods between roughly four months and two years of age, and this is likely to be stable across childhood and adulthood [178].

Thus, the introduction of whole grains in infancy offers a great opportunity for the acceptance of whole grains across the entire lifespan. Also, this period generates a favorable circumstance to introduce less sweet non-hydrolyzed infant cereals instead of their hydrolyzed counterparts. Sensory learning techniques that parents or caregivers can use to encourage familiarity include starting with small bites (preferably without any added flavors), aiming for at least 10 exposures, and encouraging repeated tasting at a regular interval [179]. Taste preferences often form the greatest challenge in the development of whole grain food products [180].

## 5. Conclusions

Infant cereals are one of the first foods given during complementary feeding. They play an important role in the early stage of life by providing the infant with energy, macronutrients, vitamins, minerals, bioactive compounds, and non-digestible carbohydrates that stimulate the gut microbiota. The existing evidence reviewed in this article suggests that whole grains are more beneficial for health compared to refined cereals. Whole grains are rich in compounds that induce several mechanisms that aid in reducing the risk of non-communicable diseases. With respect to the beneficial impact of whole grains in adults, the low intake in children and adolescents, and the consumers’ healthy perception of whole grains, it can be concluded that the incorporation of whole grains in infant cereals is a great opportunity for the future. However, there are some important issues that retailers, manufacturers, researchers, and policy makers need to address. Consistent and unified whole grain recommendations for infants and young children under two years of age are still lacking, which is probably due the scarcity of research that has been done in this vulnerable age group. Another barrier is the inconsistent product labeling and the difficulty that consumers have identifying whole grain foods. Manufacturers also need to deal with natural and process contaminants in whole grains. However, the hardest challenge that comes along with the production of whole grain food products and its ultimate acceptance by consumers is sensory appeal. Although people often prefer refined cereals, the gradual or unknown replacement of refined whole grains does not seem to negatively influence sensory acceptability, even in infants. The complementary feeding period is an important time for shaping the infant’s food preferences, eating skills, and habits, and therefore, it is the right time to introduce whole grain infant cereals for the acceptance of whole grains across the entire lifespan.

## Figures and Tables

**Table 1:** Examples of cereal recommendations for infants and young children.

Country/Region and Organization	Wording, Recommendation or Guideline
Australia: National Health and Medical Research Council (NHMRC) [27]	Infant cereals, dry, mixed grain, fortified Six to 12 months: seven serves per week, one serve weighs 20 g
Europe: European Food Safety Authority (EFSA) [22]	“It should be noted that for formula-fed infants and some breast-fed infants after four to six months of age, an intake equivalent to this value (0.3 mg per day iron from breast milk) is not sufficient to maintain iron status within the normal range”.
Europe: Domellöf et al.; Fewtrell et al. (on behalf of the European Society for Paediatric Gastroenterology, Hepatology and Nutrition, or ESPGHAN) [15,21]	“There may be some beneficial effects on iron stores of introducing complementary food alongside breast-feeding from four months. Iron-rich complementary foods are recommended, these include iron-fortified foods such as cereals. Gluten may be introduced between four and 12 months”.
France: Manger Bouger & Ministère des Solidarités et de la Santé [31]	0–4 months: no cereal intake
	5–6 months: cereals without gluten
	>7 months: cereals with gluten
New Zealand: Ministry of Health [25,26]	0–6 months: no cereal intake 6–7 months: iron-fortified cereals, puréed plain rice 7–8 months: age-appropriate infant cereals 8–12 months: breakfast cereals such as porridge, wheat biscuits (iron-fortified), infant muesli 12–24 months: all previously listed cereals 2–5 years: “At least four servings of cereals per day. Increasing whole grain options as children age”.
Spain: Asociación Española de Pediatría (AEP) [29]; Peña Quintana et al. (AEP) [30]; Dalmau Serra & Moreno Villares (AEP) [32]	< 5–6 months: cereals without gluten > 5–6 months: cereals with gluten 12 months: 57 g of cereals per day 24–36 months: 86 g of cereals per day “Fortified or whole grain (preferred) cereals, bread and pastas are suggested”.
US: American Academy of Pediatrics (AAP) [23,24]	“A baby’s digestive system is not thought to be well prepared to process cereals until about six months of age. When he is old enough to digest cereal, he should also be ready to eat it from a spoon”. “Baby cereals are available premixed in individual containers or dry, to which you can add breast milk, formula, or water. Whichever type of cereal you use, make sure that it is made for babies and iron fortified”.
US: Food and Nutrition Service, United States Department of Agriculture (USDA) [28]	Breakfast, lunch, supper, or snack 6–11 months: 0–4 tablespoons of iron-fortified infant cereals or iron-fortified ready-to-eat breakfast cereals (in case of a snack) “A serving of this component is required when the infant is developmentally ready to accept it. A serving of grains must be whole grain rich, enriched meal, or enriched flour. Breakfast cereals must contain no more than 6 g of sugar per dry ounce (no more than 21 g sucrose and other sugars per 100 g of dry cereal)”.

**Table 2:** Nutritional composition differences between whole and refined wheat flour, per 100 g [71].

Nutrient	Whole Wheat Flour	Refined Wheat Flour (75% Extraction)
Carbohydrates, g (% of energy)	62 (75.6)	71 (80.6)
Protein, g (% of energy)	10 (12.2)	12.6 (14.3)
Fat, g (% of energy)	2 (5.5)	1.1 (2.8)
Dietary fiber, g	11	4
Vitamin B1, mg	0.4	0.07
Vitamin B2, mg	0.15	0.04
Vitamin B3, mg	5.7	1
Vitamin B6, mg	0.35	0.12
Vitamin B9, mg	0.037	0.022
Vitamin E, mg	1.4	0.4
Vitamin K, mg	0.019	0.008
Iron, mg	4	0.8
Zinc, mg	2.9	0.64
Magnesium, mg	124	20
Sodium, mg	5	2
Potassium, mg	250	156
Phosphorus, mg	370	103

**Table 3:** Examples of whole grain recommendations for infants and young children.

Country/Region and Organization	Wording, Recommendation, or Guideline
Australia: National Health and Medical Research Council (NHMRC) [27]	13–23 months: - Whole grain or higher fiber cereals/grains: 16 serves * per week - Refined or lower fiber cereals/grains **: 8.5 serves * per week 24–36 months: - Whole grain or higher fiber cereals/grains: 19 serves * per week - Refined or lower fiber cereals/grains **: Nine serves * per week
Spain: Varea Calderón et al. [129]	<24 months: “It is necessary to consume four to six servings of cereals per day to meet the dietary requirements; moreover, half of these servings should be whole grain to meet the fiber requirements”.
US: American Academy Pediatrics (AAP) [23,24]; American Heart Association (AHA) [131] and Gidding et al. [133]	12 months: - Two ounces of cereals per day - Make sure half of the amount is whole grain 12–24 months: - Three ounces of cereals per day - Make sure half of the amount is whole grain “Serve whole grain breads and cereals rather than refined grain products. Look for *whole grain* as the first ingredient on the food label”.
US: Food and Nutrition Service, US Department of Agriculture (USDA) [28]	“At least one serving per day, across all eating occasions, must be whole grain or whole grain-rich. Grain-based desserts do not count towards meeting the grains requirement”. 12–24 months: Breakfast (minimum amount to be served) - ½ slice of whole grain-rich or enriched bread or; - ½ serving of whole grain-rich or enriched bread product, such as biscuit, roll, or muffin, or; - ¼ cup of whole grain-rich, enriched, or fortified cooked breakfast cereal, cereal grain, and/or pasta Lunch and supper (minimum amount to be served) - ½ slice of whole grain-rich or enriched bread or; - ½ serving of whole grain-rich or enriched bread product, such as biscuit, roll, or muffin or; - ¼ cup of whole grain-rich, enriched, or fortified cereal, cereal grain, and/or pasta Optional best practices that providers may choose to implement to make further nutritional improvements to the meals they serve: “The Institute of Medicine (IOM) recommended that at least half of all grains served are whole grain-rich. To meet this goal, providers are encouraged to prepare at least two servings of whole grain-rich grains each day. This is an increase from the required one serving of whole grain-rich grains per day”.
US: Healthy Eating Research, Pérez-Escamilla et al. [130]	Six to 12 months: “What your baby eats at around nine months is indicative of what she/he will like to eat when school-aged. Offer your baby a variety of vegetables and fruits and whole grain products (e.g., brown rice, whole grain cereals)”. 12–24 months: “Offer your toddler whole grain food, such as whole wheat bread, whole wheat pasta, maize tortillas, or brown rice. These food items are rich in fiber, which is often missing from children’s diets. Offer ½ to one slice of whole grain bread, or ¼ to ½ cup of whole grain cereal or pasta at most meals and snacks”.
US: US Department of Health and Human Services (HHS) and US Department of Agriculture (USDA) [45]	12–36 months: - Whole grain: 1.5 to 2.5-ounce equivalents *** or above - At least half of total grain consumption is whole grain

* One serve is equivalent to 40 g of bread. ** Refined or lower fiber cereals were included as a group for cultural reasons; whole grain or higher fiber can replace these if preferred. *** In general, one slice of bread, one cup of ready-to-eat cereal, or ½ cup of cooked rice, cooked pasta, or cooked cereal can be considered as a one-ounce equivalent.

**Table 4:** Main findings of studies evaluating consumers’ sensory acceptability of whole grain foods.

Author, Year	Country	Age	Main Results
Brownlee et al. 2013 [164]	UK	+18 years	“Whole grain consumption was significantly higher in participants who were provided with whole grain foods throughout the intervention period compared with the control group that was not provided with whole grain foods (approximately doubled, *p* < 0.001) and compared to baseline”.
Chan et al. 2008 [166]	US	6–11 years	“There was no difference in children’s consumption of the 50:50 blend pizza (50% whole grain and 50% refined grain) compared to the 100% refined counterpart (mean consumption 106 ± 4 g of 50:50 pizza compared to 100 ± 2 g of refined pizza)”.
Haro-Vicente et al. 2017 [169]	Spain	4–24 months	“Overall acceptability for infant cereals with whole grain and refined cereals was very similar both for infants (2.30 ± 0.12 and 2.32 ± 0.11, *p* = 0.606) and parents (6.1 ± 0.8 and 6.0 ± 0.9, *p* = 0.494). Sensory evaluation of the color, aroma, taste, and texture by parents indicated no significant difference between both types of infant cereals (all *p* > 0.05)”.
Kuznesof et al. 2012 [112]	UK	18–65 years	“Many participants expressed surprise at liking the taste of whole grain foods that they had either prejudged to be ‘tasteless’ or recalled (on the basis of a previous eating experience) to taste inferior to alternatives. A preference for certain whole grain foods was established over time”.
Magalis et al. 2016 [116]	UK	18–19 years	“Both refined rice and refined pasta were significantly more well-liked than their whole grain counterparts for all sensory attributes (*p* ≤ 0.05). For tortillas and bread, the whole wheat and refined wheat samples were similarly well-liked (*p* > 0.05)”.
Mellette et al. 2018 [165]	US	18–24 years	“Respondents liked all muffin formulations (muffins containing 50%, 75%, and 100% whole wheat flour) similarly for appearance, taste, texture, and overall liking. After the whole grain content of each muffin was revealed, 66% of students increased their liking of the muffin containing 100% whole wheat flour”.
Neo and Brownlee, 2017 [163]	Singapore	21–26 years	“The whole grain familiarization period did not alter the taste expectations of the consumers, but it did manage to increase acceptance for four of the whole grain products tested (*p* < 0.001 for oatmeal cookie, granola bar, and muesli, *p* < 0.05 for wheat biscuit breakfast cereal”.
Rosen et al. 2008 [167]	US	Kindergarten–6th grade children	“Mean consumption of buns and rolls at the baseline for both schools (0% whole wheat) was ~75%. Intake of bread products did not differ significantly from the baseline level up to the 59% level of red whole wheat and 45% of the white whole wheat. The range of consumption for dinner rolls made with red whole wheat flour was 57% to 77%, while white whole wheat flour was 50% to 78%, indicating that grain bread products may be more acceptable, with a total whole grain flour content approaching 75%”.
Toma et al. 2009 [168]	US	Kindergarten–6th grade children	“No significant differences (*p* > 0.05) in consumption between control products (products with refined flour) and test products (burritos and cookies containing 51% and 100% whole grain, respectively) were found”.
